# Identifying rare variants using a Bayesian regression approach

**DOI:** 10.1186/1753-6561-5-S9-S99

**Published:** 2011-11-29

**Authors:** Aimin Yan, Nan M Laird, Cheng Li

**Affiliations:** 1 Department of Biostatistics, Harvard School of Public Health, 677 Huntington Avenue, Boston, MA 02115, USA; 2 Department of Biostatistics and Computational Biology, Dana-Farber Cancer Institute, 44 Binney Street, Mailstop CLS11007, Boston, MA 02115, USA

## Abstract

Recent advances in next-generation sequencing technologies have made it possible to generate large amounts of sequence data with rare variants in a cost-effective way. Statistical methods that test variants individually are underpowered to detect rare variants, so it is desirable to perform association analysis of rare variants by combining the information from all variants. In this study, we use a Bayesian regression method to model all variants simultaneously to identify rare variants in a data set from Genetic Analysis Workshop 17. We studied the association between the quantitative risk traits Q1, Q2, and Q4 and the single-nucleotide polymorphisms and identified several positive single-nucleotide polymorphisms for traits Q1 and Q2. However, the model also generated several apparent false positives and missed many true positives, suggesting that there is room for improvement in this model.

## Background

Rare variants are genetic variants that have a minor allele frequency (MAF) less than 1%. Many previous studies have suggested that rare variants generally have larger effects on a trait than common variants. Therefore identification of rare variants has become an important research topic in recent genome-wide association studies. Several statistical approaches have been developed to tackle this problem. These methods include the weighted sum statistic [1], combined multivariate and collapsing [2], the comparison of rare variants found exclusively in case subjects to those found only in control subjects [3,4], and the kernel-based adaptive cluster [5]. Overall, the results observed from these studies indicate that multiple rare variants collectively contribute to the variations of the trait, suggesting that it is desirable to use all variants together to identify the associated genetic variants with a given phenotype.

Bayesian regression models have been used in animal breeding to predict breeding values based on all available single-nucleotide polymorphisms (SNPs) [6]. Many extensions of Bayesian regression models in this field have been discussed by Gianola et al. [7]. Bayesian stochastic variable selection methods have also provided an alternative approach to genome-wide association studies [8,9]. Srivastava and Chen [10] compared the performance of a Bayesian stochastic variable selection method with that of a penalized sparse regression method and demonstrated that the Bayesian stochastic variable selection outperformed the sparse regression and also the single-SNP-based method. Yi and Zhi [11], in a recent study, used Bayesian stochastic variable selection for the identification of rare variants. However, the studies by Srivastava and Chen [10] and Yi and Zhi [11] did not estimate the probability that the SNP will be associated with the phenotype given the data.

In the current study, we model common variants and rare variants simultaneously using a Bayesian stochastic variable selection method. We calculate the regression coefficient and posterior probability of association of each SNP and use them to measure the association between each SNP and the given trait. The difference between our method and those of Srivastava and Chen [10] and Yi and Zhi [11] is that we estimate the posterior probability that the SNP will be associated with the phenotype and use that probability to estimate the number of SNPs associated with the given trait. We apply this method to study the association for the quantitative risk factors Q1, Q2, and Q4 in the Genetic Analysis Workshop 17 (GAW17) data and successfully identify several SNPs associated with the Q1 and Q2 traits.

## Methods

### Overall model

First, let us introduce the model and some notation. The model is:(1)

where , *n* is the number of individuals, *p* is the number of SNPs, **y** is an *n* × 1 phenotype vector, **X** is an *n* × *p* matrix with entries being 0, 1, and 2 encoded for the genotypes *AA*, *AB*, and *BB*, respectively, **θ** is a *p* × 1 latent variable vector with entries being 0 and 1 to perform variable selection, and **α** is a *p* × 1 regression coefficient vector. **θ** ∘ **α** indicates the element-wise product between **θ** and **α**. If *θ_j_* = 1, then *α_j_* (SNP *j*) is included in the model; if *θ_j_* = 0, then *α_j_* is excluded from the model. *P*(*θ_j_* = 1) = *π* is the prior probability that the SNP will be associated with the phenotype in question, where *π* is the same for all SNPs. We assume that the prior probability for *k* is Binomial(*B*(*p*, *π*)), where *k* is the number of SNPs that are associated with a phenotype. *α_j_* is normally distributed with mean 0 and variance , and  is the scaled inverse chi-square distribution with scale parameter *S_α_* and degrees of freedom *v_α_*.  follows the scaled inverse chi-square distribution with scale parameter *S_ε_* and degrees of freedom *v_ε_*.

### Posterior estimations

Based on Eq. (1), one can obtain the full conditional probability functions (FCPFs) for the parameters (derivations not shown). The FCPF for  is the scaled inverse chi-square distribution with scale parameter:(2)

and degrees of freedom *v_ε_* + *n*, where:(3)

In expression (2), **x***_j_* is an *n* × 1 vector that corresponds to the SNP *j*.

The FCPF for *μ* is the normal distribution with mean:(4)

and variance .

The FCPF for *α_j_* is the normal distribution with mean  and variance , where:(5)

and:(6)

It is clear that the  is conditional on all other .

The FCPF for  of each locus is the scale inverse chi-square distribution with scale parameter:(7)

and degrees of freedom , where  is a vector in which the  is not 0. To obtain , we need to decide whether each SNP should be included in the model or not. To make this decision, we need to calculate:(8)

In expression (8),  indicates that the *α_j_* are not in the model in the *s*th Markov chain Monte Carlo (MCMC) iteration; and  is the likelihood that the *α_j_* are not in the model in the *s*th MCMC iteration and is given by:(9)

In addition,  indicates that the *α_j_* are in the model in the *s*th MCMC iteration; and  is the likelihood that the *α_j_* are in the model in the *s*th MCMC iteration and is given by:(10)

In expression (8), , and the posterior distribution for *π* is the Beta distribution (Beta). From these likelihoods and the sampled *π*, we compute the value of expression (8), and use this value to determine whether  is 1 or 0. Posterior estimations are based on the samples from the given FCPFs using MCMC sampling.

### MCMC sampling

MCMC sampling works as follows. For each MCMC iteration, we first sample  from its FCPF and *μ* from its FCPF. Next, for *α*_1_, *α*_2_, *α*_3_, …, *α_j_*, …, *α_p_*, we sample from the FCPF for *α_j_*. Whether *α_j_* is included in the model or not is determined, and  is updated by .  is estimated. Next we sample from the FCPF for . Finally, we sample *π* from Beta.

We performed 15,000 MCMC iterations and used the first 1,000 iterations as the burn-in period. The inclusion probability for *α_j_* is based on the proportion of *θ_j_* = 1 in all the MCMC samples after the burn-in period. This probability is used as the posterior probability of association (PPA) for each SNP.

### Data set

The GAW17 data set includes 697 unrelated individuals; each individual has 24,487 SNPs. The MAFs of the SNPs range from 0.0717% to 49.9283% [12]. Our analysis is based on quantitative traits Q1, Q2, and Q4. The GAW17 answers show that Q1 is associated with 39 SNPs in 9 genes from the vascular endothelial growth factor (VEGF) pathway, that Q2 is influenced by 72 SNPs in 13 genes related to cardiovascular risk and inflammation, and that Q4 is not affected by any of the available SNPs. There are 200 data replications for each trait. We perform an analysis for each replication and obtain the average regression coefficients and PPAs for each SNP from the 200 data replications. We then use the different cutoffs of the regression coefficients and PPAs to compute a series of true-positive rates (TPRs) and false-positive rates (FPRs). We use the receiver operating characteristic (ROC) to compare the TPR and the FPR as the cutoffs change and the area under the curve (AUC) to measure the performance of the model.

## Results and discussion

We analyzed the association between quantitative traits Q1, Q2, and Q4 and the SNPs in the GAW17 data. For each trait, we assigned a relative rank for each SNP on the basis of the sorting of the absolute values of the average regression coefficients and PPAs of all SNPs in decreasing order. In our model, we estimated the number of SNPs associated with a trait. Using this number (), we identified the SNPs whose ranks are within the range of this number.

For the Q1 trait, the range of the estimated number of SNPs associated with Q1 is 3 to 8. Based on this range, the SNPs whose average regression coefficients and PPAs are within the top eight rankings are considered associated with Q1 (Table 1). The ranks of C13S431, C13S522, C13S523, C1S6533, and C4S1884 are within the top eight. The GAW17 answers confirmed that all five SNPs are truly associated with Q1. C13S431, C13S522, and C13S523 are located in gene *FLT1*, C1S6533 is located in gene *ARNT*, and C4S1884 is located in gene *KDR*.

**Table 1:** Association analysis for trait Q1

Gene	SNP	Regression coefficient	PPA	MAF
*FLT1*	C13S431	2.501 (2)	0.243 (3)	0.017217
	C13S522	2.188 (3)	0.264 (2)	0.027977
	C13S523	9.027 (1)	0.998 (1)	0.066714
*ARNT*	C1S6533	0.478 (6)	0.043 (4)	0.011478
*KDR*	C4S1884	0.126 (42)	0.016 (8)	0.020803

The third and fourth columns are the average regression coefficients and posterior probabilities of association (PPAs) out of 200 replications, respectively. The numbers in parentheses for the regression coefficients indicate the rank of the SNP based on sorting the absolute values of average regression coefficients in decreasing order. The numbers in parentheses for the PPAs indicate the rank of the SNP based on sorting the PPAs in decreasing order. We use the same notation in Table 2.

**Table 2:** Association analysis for trait Q2

Gene	SNP	Regression coefficient	PPA	MAF
*VNN1*	C6S5380	0.251 (1)	0.077 (1)	0.170732
*VNN3*	C6S5449	0.194 (2)	0.015 (3)	0.010043
	C6S5441	0.038 (25)	0.010 (4)	0.098278
*LPL*	C8S442	0.152 (5)	0.016 (2)	0.015782
*SIRT1*	C10S3050	0.170 (3)	0.008 (6)	0.002152

See Table 1 notes for an explanation of the notation.

For the Q2 trait, the range of the estimated number of SNPs associated with the Q2 is 2 to 6. Based on this range, the SNPs whose average regression coefficients and PPAs are within the top six rankings are considered associated with Q2 (Table 2). We found that the ranks of C6S5380, C6S5449, C6S5441, C8S442, and C10S3050 are within the top six. C6S5380 is located in gene *VNN1*, C6S5449 and C6S5441 are in gene *VNN3*, C8S442 is in gene *LPL*, and C10S3050 is a rare variant in gene *SIRT1* with a MAF = 0.002152. The GAW17 answers confirmed that all five SNPs are truly associated with Q2. In our analysis, C10S3051 is also identified as being associated with Q2. Compared with the GAW17 answers, this finding is a false-positive association. However, we found that C10S3051 is a synonymous mutation and is identical to C10S3050. The positions of the two SNPs are close together, suggesting that the two SNPs may be in high linkage disequilibrium.

For the Q4 trait, the range of the estimated number of SNPs associated with Q4 is 3 to 6. Based on this range, the SNPs whose average regression coefficients and PPAs are within the top six rankings are considered associated with Q4. Compared with the GAW17 answers, these SNPs are false positives. We observed that the correlation coefficient between Q1 and Q4 is −0.293 and that there are 39 SNPs that are associated with Q1, which could be the reason for these false positives.

The results for Q1 and Q2 demonstrate that our model identified several true SNPs associated with Q1 and Q2 but missed many true positives for these two traits. To assess the model’s performance for identifying rare variants, based on the association results using all SNPs, we extract the SNPs with a MAF less than 1% and calculate the AUCs using all SNPs and using the rare variants only for Q1 and Q2. Table 3 shows that the AUCs using all SNPs range from 0.774 to 0.808; the AUCs using only the rare variants range from 0.699 to 0.724. We obtained a reasonable power to detect rare variants using this model. However, it is obvious that the power of our model to identify rare variants is less than the power to identify common variants. These results could be due to the small effects of SNPs with lower MAFs, and our model shrinks their regression coefficients to 0.

**Table 3:** AUC of the model using all SNPs and the rare variants only for traits Q1 and Q2

Trait	AUC based on regression coefficients	AUC based on PPAs
Q1	0.791 (all SNPs )	0.808 (all SNPs)
	0.699 (RV only)	0.724 (RV only)
Q2	0.776 (all SNPs)	0.774 (all SNPs)
	0.724 (RV only)	0.718 (RV only)

“All SNPs” indicates our analysis based on all SNPs; “RV only” indicates our analysis based on only the rare variants (MAF < 0.01).

Several other factors could also have played a role in causing the false negatives and false positives. First, we observed that there is an outlier for the Q1 trait. Several studies have shown that removing this outlier might increase the detection power. Our analyses did not consider these outliers, so we expected that we could increase the power by removing the outliers in the subsequent analyses. Second, the structure information of the SNPs was not included in the model, although all SNPs were modeled simultaneously. Many studies have shown that collapsing SNPs into blocks based on linkage disequilibrium, a gene, or a biological pathway can increase the power to detect associations. In a future study, we plan to model the correlations between the SNPs or to collapse the SNPs into blocks first and then apply this model to the blocks to see whether the detection power of this method can be increased.

## Conclusions

In the present study, we modeled all SNPs simultaneously to study the association between the SNPs and the quantitative risk traits Q1, Q2, and Q4 using a Bayesian regression method. Some true associated SNPs for Q1 and Q2 were identified using this method. However, our model missed many true positives and generated several false positives, suggesting that there is room for improvement.

## Competing interests

The authors declare that they have no competing interests.

## Authors’ contributions

AY performed data analysis and drafted the manuscript. NML and CL helped with data analysis and manuscript writing. All authors read and approved the final manuscript.
